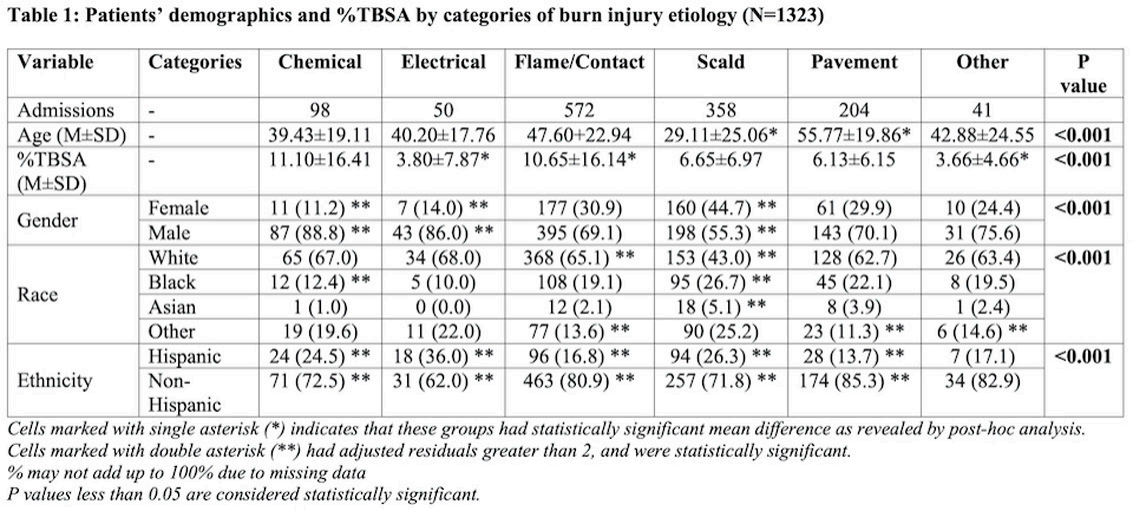# 34 Evaluation of Socioeconomic Status and Burn Injuries: A Retrospective Review From a Desert Burn Center

**DOI:** 10.1093/jbcr/irad045.008

**Published:** 2023-05-15

**Authors:** Samuel Cohler, Paul Chestovich, Syed Saquib, Kavita Batra

**Affiliations:** Kirk Kerkorian School of Medicine at UNLV, Las Vegas, Nevada; ; Kirk Kerkorian School of Medicine at UNLV, Las Vegas, Nevada; Kirk Kerkorian School of Medicine at UNLV/UMC Lions Burn Care Center, Las Vegas, Nevada; Kirk Kerkorian School of Medicine at UNLV, Las Vegas, Nevada; Kirk Kerkorian School of Medicine at UNLV, Las Vegas, Nevada; ; Kirk Kerkorian School of Medicine at UNLV, Las Vegas, Nevada; Kirk Kerkorian School of Medicine at UNLV/UMC Lions Burn Care Center, Las Vegas, Nevada; Kirk Kerkorian School of Medicine at UNLV, Las Vegas, Nevada; Kirk Kerkorian School of Medicine at UNLV, Las Vegas, Nevada; ; Kirk Kerkorian School of Medicine at UNLV, Las Vegas, Nevada; Kirk Kerkorian School of Medicine at UNLV/UMC Lions Burn Care Center, Las Vegas, Nevada; Kirk Kerkorian School of Medicine at UNLV, Las Vegas, Nevada; Kirk Kerkorian School of Medicine at UNLV, Las Vegas, Nevada; ; Kirk Kerkorian School of Medicine at UNLV, Las Vegas, Nevada; Kirk Kerkorian School of Medicine at UNLV/UMC Lions Burn Care Center, Las Vegas, Nevada; Kirk Kerkorian School of Medicine at UNLV, Las Vegas, Nevada

## Abstract

**Introduction:**

Incidence of burn injury rate appears to be affected by socioeconomic status (SES). Previous studies have indicated that patients with low SES have an increased risk of unintentional burn injuries. However, this association has not been explored robustly in the Southwest United States. This study aims to investigate the relationship between SES and burn injuries amongst patients in the Southwest United States.

**Methods:**

A retrospective registry review was performed on all burn injury admissions to our ABA verified burn center from January 1, 2017 to December 31, 2021. Their zip code was then inputted into the Distressed Communities Index (DCI). This is a national database that stratifies communities by zip code into quintiles, indicating overall distress. The DCI was used as a measure of SES as it reflects employment status, education level, poverty rate, median income, business growth, and housing vacancies. Patients who did not report an accurate residential zip code or reported a zip code not included in the DCI were excluded. Many clinical variables were reviewed within each quintile including age, gender, total body surface area (TBSA), ICU days, hospital length of stay (LOS), ventilator days, surgical interventions, and discharge disposition. Student’s t-test and Chi-squared tests were used to compare groups, and statistical significance was defined at p < 0.05.

**Results:**

A total of 1,323 patients were identified in the study period. The most distressed quintile accounted for 481 (36.4%) admissions. Flame and contact burns were the most common etiology overall (46.5%), as well as within each quintile (Figure 1). The distribution of burn injury etiology was statistically significant by age, %TBSA, gender, race, and ethnicity (p < 0.001, Table 1); however, it was not significant by SES (p = 0.202). Burn severity, categorized as less than or equal to second degree burns, or greater than or equal to third degree burns, showed significance when compared to age, LOS, ICU days, ventilator days, and total surgical interventions performed (p < 0.001). However, the association between SES and burn severity was not found to be significant (p = 0.886).

**Conclusions:**

The two most distressed quintiles represented over half of the burn admissions. A lower SES did not contribute to increased burn severity or utilization of healthcare resources. This study highlighted differences in burn injuries between age, gender, race, and ethnicity.

**Applicability of Research to Practice:**

Identifying communities at increased risk for burn injuries can help with public health initiatives, focused on awareness and prevention of these injuries.